# Presepsin as a Diagnostic and Prognostic Biomarker of Sepsis-Associated Acute Kidney Injury: A Scoping Review of Clinical Evidence

**DOI:** 10.3390/jcm14196970

**Published:** 2025-10-01

**Authors:** Edmilson Leal Bastos de Moura, Dilson Palhares Ferreira, Rinaldo Wellerson Pereira

**Affiliations:** 1 Health Sciences Doctoral Program, University of Brasília (UnB), Brasilia 70910-900, Brazil; 2 School of Health Sciences, Distrito Federal University (UnDF), Brasilia 70710-907, Brazil; palhares.dilson@gmail.com; 3 Genomic Sciences and Biotechnology Graduate Program, Catholic University of Brasilia, Brasilia 71966-700, Brazil; rinaldo@p.ucb.br

**Keywords:** presepsin, sCD14-ST, acute kidney injury, renal injury, sepsis, septic shock, sepsis-associated acute kidney injury

## Abstract

Sepsis is a complex clinical syndrome associated with high morbidity and mortality and organ dysfunction, most notably acute kidney injury. Early recognition determines crucial clinical decisions for septic individuals. This rapid diagnosis depends on the accuracy of biomarkers in the context of coexisting renal dysfunction. In this context, the value of presepsin has been investigated and challenged for a decade, with no definitive answers. This scoping review aims to evaluate the existing evidence regarding the accuracy of presepsin as a diagnostic and prognostic biomarker for sepsis-associated acute kidney injury (SA-AKI). We obtained 130 articles by searching for references in databases (PubMed/Medline, Web of Science, Embase, and Scopus) following the PRISMA-ScR guidelines. Sequential selection by three independent readers resulted in nine references retained for full analysis. Presepsin demonstrated good diagnostic and prognostic accuracy in patients with AKI, based on observations in small patient groups; however, it requires specific cutoff values, whose determination depends on new controlled and randomized studies.

## 1. Introduction

Sepsis is a medical emergency that affects approximately 1.7 million adults in the United States annually, contributing to more than 250,000 deaths [1]. It affects approximately 49 million people worldwide [2], accounting for 20% of deaths [3]. Various studies estimate that sepsis is present in 30% to 50% of hospitalizations that result in death [1]. Ye et al. [4] highlighted that sepsis-induced acute kidney injury (SA-AKI), a common complication of sepsis, significantly increases patient mortality, prolongs hospital stay, and raises treatment costs, with risk factors such as advanced age, severity of AKI, hypoalbuminemia, late antibiotic administration, and elevated bilirubin levels.

The diagnosis of sepsis is clinical and must be made early to mitigate its adverse outcomes (e.g., septic shock, multiple organ failure, and permanent organ damage) and high mortality. Therefore, global efforts have been made to optimize its diagnosis, risk stratification, treatment, and prognosis.

In this scenario of shortening the diagnostic process of sepsis, biomarkers have gained notoriety, with numerous studies supporting their use in risk stratification, etiology identification, severity assessment, and the prediction of duration and recovery from AKI. A recent study observed a significant increase in publications involving biomarkers in sepsis [5].

For this purpose, there are other promising biomarkers for diagnosing SA-AKI, such as neutrophil gelatinase-associated lipocalin (NGAL) or proenkephalin (PENK). NGAL is correlated with nephrotoxic insults, but conditions such as sepsis, chronic obstructive pulmonary disease, cardiac dysfunction, diabetes, and hypertension can interfere with its measurement. PENK may play a regulatory role in renal function and is an independent predictor of severe AKI, but it may be confounded by the coexistence of heart failure and transplantation [6].

Presepsin (PSP) has been considered a candidate due to its high specificity for infections and the number of publications exploring its characteristics. It is a molecule of approximately 13 kDa, an N-terminal fragment of the CD14 differentiation marker protein cluster, which exists in soluble (sCD14) or membrane-bound (mCD14) forms [7]. CD14 is a transmembrane glycoprotein that is part of the toll-like receptor family and recognizes various ligands from both Gram-positive and Gram-negative bacteria, such as lipopolysaccharide (LPS), activating intracellular signaling pathways and immune response [8]. PSP is a soluble fraction of the LPS receptor and can normally be detected in the plasma of infection-free healthy individuals at low concentrations [8].

Initially described as a biomarker for sepsis in 2005 [9], it was considered to have an early onset (rising before C-reactive protein, which begins to rise 4–6 h after the onset of infection, with a half-life of 24 h, peaking at 48–72 h) [7], typically low in healthy adults < 0.3 mg/dL, often exceeding 50 mg/dL in severe infections).

Although it is considered of special importance in bacterial sepsis [10,11], CD14 is not produced primarily by monocytes and macrophages [12], but also occurs in non-hematopoietic cells after the induction of mRNA transcription in pulmonary, renal, and hepatic epithelial cells, as well as endothelial, microglial, and vascular myocytes, secondary to endotoxemia [13].

No reliable biomarker has been identified as ideal in clinical sepsis settings, such as emergency departments and intensive care units. PSP was weakly recommended, only as a supplement to clinical observation, in the 2020 Japanese Clinical Guidelines for the Management of Sepsis and Septic Shock [14]. However, its routine use has been compromised, as PSP has characteristics that allow it to be filtered through the glomeruli, reabsorbed, and catabolized within the proximal tubular cells, urging careful interpretation in patients with kidney disease; that is, PSP levels and the glomerular filtration rate (GFR) are inversely correlated [15]. Thus, PSP’s value as a reliable indicator for sepsis in individuals in advanced stages of AKI has been challenged, considering that there is no statistical difference between septic and non-septic patients, according to some authors [16].

Currently, some approved therapies can improve sepsis-induced kidney injury (SA-AKI), such as continuous renal replacement therapy, mainly focusing on supporting the patient’s kidney failure; broad-spectrum antibiotics to treat the underlying sepsis; and vasopressors in the treatment of shock, controlling possible complications of sepsis. However, there is still no specific treatment for this disorder.

This scoping review aims to evaluate the scientific evidence that assesses the efficacy of presepsin as a diagnostic biomarker in clinical situations where sepsis and AKI coexist, that is, in SA-AKI.

## 2. Background—Thematic Analysis

A brief, non-exhaustive discussion of the topics related to the understanding of this review is given below, allowing comprehension of the state of the art, consensus, and controversies involving the subject, based on some relevant publications.

### 2.1. SA-AKI

SA-AKI, previously termed sepsis-related AKI [17] or sepsis-induced AKI [18], has been studied for decades. Although its multifactorial origin is unanimous among authors, there is no agreement regarding the etiological agents. Studies have addressed the critical illness scenario, pointing to possible interventions that may prevent or attenuate the condition’s onset, such as anticoagulant therapy, early resuscitation, treatment of hyperglycemia, and shortened use of ventilatory support [17].

SA-AKI has a unique pathophysiology that differentiates it from AKI with other etiologies; in the former, the paradigm of ischemia producing tubular necrosis is insufficient to account for all the observed phenomena. Experimental studies support this new concept, showing unchanged renal medullary and cortical flow in hyperdynamic septic situations [19] or endotoxemia [20].

A unifying theory proposed by Gomez and colleagues [21] suggests an adaptive origin to the renal tubular epithelial cell response, driven by mitochondria, justifying its unique clinical phenotype. It, therefore, postulates that other mechanisms are relevant, such as microvascular dysfunction (associated with low flow and tubular oxidative stress), downregulation of tubular metabolism, and reformulation of priority cellular functions (oxidative stress, inflammation, and induction of an adaptive response, involving the reprioritization of energy consumption, mitophagy, and cell cycle arrest).

In an editorial written by Pettila and Bellomo in 2014 [22], similar pathophysiological mechanisms were also proposed, involving abnormalities in microcirculatory flow, inflammation, and cellular bioenergetic adaptive responses to injury. It emphasizes, however, that there are numerous gaps in our knowledge on the subject, despite several advances achieved.

Its pathophysiology was addressed in the consensus of the 28th Acute Illness Quality Initiative (ADQI) held in 2022 [23]. Such particularities involve systemic inflammation, cardiovascular depression, immunomodulation, mitochondrial dysfunction, and metabolic reprogramming, acting synergistically but uniquely in each person, justifying their specific conceptual approach [23,24].

### 2.2. PSP Kinetics and Serial Measurements

Fischer and colleagues describe a correlation between 28-day mortality and variations in serum PSP levels above 500 ng/mL [25]. Similarly, blood levels obtained on the sixth day of hospitalization that showed a 50% reduction compared with admission levels were predictors of better survival in septic individuals [26].

Serial PSP measurement has been identified as valuable in the diagnosis of sepsis and may be advantageous over isolated measurements. In this regard, Masson and colleagues [10] obtained samples on days 1, 2, and 7 after admission, revealing statistically higher values in non-survivors (*p* < 0.005).

Even in patients with renal dysfunction, Kotera and colleagues [27] recommend that sequential measurements can be beneficial and should be favored for discriminating between septic and non-septic patients.

### 2.3. PSP in Renal Physiology and Pathophysiology

PSP plasma levels are detectable within the first 6 h after the onset of infection, peaking at 3 days and decreasing at 7 days [28]. In individuals with normal renal function, PSP is filtered through the glomeruli, then reabsorbed and catabolized within the proximal tubular cells, and subsequently eliminated [29]. Therefore, its serum levels should be interpreted with caution in patients with kidney disease, as they are elevated in these individuals. Nakamura and colleagues observed abnormally high serum PSP levels in patients with end-stage AKI, confirming that the kidneys are responsible for PSP clearance [16]. However, urinary PSP measurement did not show diagnostic or prognostic accuracy for SA-AKI [30].

It was demonstrated that PSP levels are positively correlated with creatinine levels and negatively correlated with the estimated glomerular filtration rate (in septic or non-septic individuals; *p* < 0.0001) [16]. Similarly, PSP levels and the glomerular filtration rate (GFR) are inversely correlated [15]. The same correlation between PSP and eGFR was found by Kang and colleagues [31], using the CKD-EPI and MDRD methods (r = −0.24 and *p* = 0.0129; r = −0.194 and *p* = 0.048, respectively) and by Han and colleagues [32], establishing a correlation with creatinine (r = 0.180; *p* 0.043) and eGFR (r = −0.251; *p* = 0.004). Miyoshi and colleagues’ study confirmed the correlation with creatinine levels represented by r = 0.834 (*p* < 0.01) [33], but they demonstrated a positive correlation with eGFR (r = 0.837; *p* < 0.01), unlike other studies.

Kobayashi and colleagues [34] found an exponential correlation between PSP concentration and renal function decline in patients with impaired renal function classified as having chronic kidney disease (CKD; eGFR 51.8 ± 28.1 mL/min/1.73 m^2^). However, considering that the GFR estimate, as well as the KDIGO classification, is based on serum creatinine levels, this parameter can be inaccurate for conjecturing about renal function. This highlights the fragility of creatinine as a biomarker and, consequently, of all scores and classifications that use it. This is because creatinine reflects a change in renal function at a later stage compared with cystatin C [33], which is much better in all CKD stages due to differences in its metabolism and less dependence on muscle mass, and reflecting kidney disorder from an earlier stage compared to creatinine [33] (a bad biomarker due to tubular abnormalities in late stages of CKD).

Kobayashi and colleagues’ study [34] observed that eGFR can be obtained using an equation based on cystatin-C levels, but this method is less widely used and widespread.

### 2.4. Diagnostic Accuracy of PSP vs. Procalcitonin (PCT)

There is controversy regarding the superiority of PSP over PCT as a biomarker in the setting of sepsis. Theoretically, PSP’s advantage over PCT is due to the former being produced in response to bacterial infections [35] in patients with sepsis or SIRS (systemic inflammatory response syndrome) compared with healthy individuals [9]. A study suggests its usefulness in patients with recurrent sepsis, where persistently elevated PSP values (>1000 pg/mL) would prevent the discontinuation of antibiotic therapy, while PCT levels normalize [36].

Some studies’ results favor PSP, such as Liu et al.’s study [37], which found an AUC value of 0.820, higher than that of PCT (AUC of 0.724), which was statistically significant (*p* < 0.01). Shozushima et al. demonstrated an AUROC value of 0.845, higher than that of PCT, at 0.652 [35].

Studies have found comparable diagnostic accuracy values between these biomarkers, as demonstrated by Ali et al. [28], with AUCs of 0.805 and 0.780 for PSP and PCT, respectively (*p* = 0.755).

Meta-analysis-derived studies, as described by Kondo and colleagues [38], showed equivalence between PSP and PCT in the early diagnostic accuracy of infection in critically ill patients, with AUROC values of 0.87 and 0.84, respectively. The group led by Wu [39] found similar results, with an AUC of 0.88 (95% CI: 0.85–0.90) for PSP, revealing good diagnostic accuracy.

However, a study showing the superiority of PCT was published by Ren and colleagues [40], with ROC curve analysis revealing a higher AUC for PCT (0.719), and PSP showing moderate accuracy (0.661; 95% CI: 0.621–0.661; *p* < 0.001).

### 2.5. PSP in Sepsis Without AKI

A meta-analysis demonstrated PSP’s moderate diagnostic accuracy (AUROC: 0.89; 95% CI: 0.84–0.94) in differentiating sepsis from non-infectious SIRS [39]. This result was corroborated in other meta-analyses, such as that conducted by Zhang and colleagues [41], with an AUROC of 0.89 (95% CI: 0.86–0.92).

The accuracy in diagnosing sepsis in individuals without AKI showed an AUC of 0.784 (95% CI: 0.683–0.860), but with no statistically significant difference from the AKI group (*p* = 0.200) [16].

A study indicated even higher levels of PSP in septic shock [40], when using a cutoff value of 2553.5 pg/mL, obtaining an AUC of 0.661 (95% CI: 0.621–0.661; *p* < 0.001), with a sensitivity of 39.6% and a specificity of 92.2%, thus indicated as an independent predictor of septic shock.

### 2.6. PSP in AKI Without Sepsis

PSP levels in patients with renal dysfunction are high even in non-infectious conditions [27]. According to Nakamura and colleagues’ analysis, groups with renal dysfunction (the failure group, according to the RIFLE classification), whether or not they had sepsis, showed no statistically significant difference in serum biomarker levels [16].

Although not specifically representative of the topic under analysis, but highlighting the impact of renal function loss on biomarker values even without the context of sepsis, we cite the work of Endo et al., which demonstrated that PSP values in two patients with chronic kidney disease without sepsis were 9036 and 1362 pg/mL, well above the threshold values of 600 pg/mL determined for that population (with a sensitivity of 87.8% and a specificity of 81.4%) [42], highlighting that data from the observation of a small number of patients are limited.

### 2.7. Cutoff Values for Sepsis Diagnosis

Different studies indicate disparate cutoff values, correlating with different sensitivity and specificity values. Godnic et al. suggest 413 pg/mL (with a sensitivity of 84.6% and a specificity of 62.5%) for the diagnosis of sepsis [43]. Other ideal cutoff values proposed by other authors for the diagnosis of sepsis include 317 pg/mL by Liu et al. (with a sensitivity of 70.8% and a specificity of 85.8%) [37], 600 pg/mL by Endo et al. (with a sensitivity of 87.8% and a specificity of 81.4% [42]), and 399 pg/mL by Shozushima and collaborators (with a sensitivity of 80.3% and a specificity of 78.5%) [35].

### 2.8. PSP in SA-AKI

PSP’s value as a reliable indicator of sepsis in patients with advanced stages of AKI has been questioned, as authors have obtained conflicting results. Nakamura et al. state that PSP levels in these patients do not show a statistical difference between septic and non-septic individuals [16].

However, another study showed divergent results. Han et al. [32] demonstrated that PSP has moderate diagnostic value in discriminating AKI at serum levels of 1390 pg/mL, revealing an AUC of 0.706 and *p* of 0.001.

### 2.9. Cutoff Values for SA-AKI Diagnosis

PSP values are variable in individuals with impaired renal function [29]. As such, authors suggest different cutoff points for the diagnosis of sepsis based on PSP levels depending on the estimated GFR, assuming ideal cutoff points of 500 pg/mL for eGFR greater than or equal to 60 mL/min/1.73 m^2^, and 1000 pg/mL when less than 60 mL/min/1.73 m^2^ [44]. Similarly, PSP limits (pg/mL) should be adjusted when using serum creatinine levels (SCr, mg/dL): for values of sCr ≤ 1.5, 1.5 < sCr ≤ 2, 2 < sCr ≤ 4, and sCr > 4, normal values ≤ 300, ≤ 500, ≤ 850, and ≤ 1800 are suggested, respectively [45].

### 2.10. PSP and Hemodialysis

Blood PSP levels are influenced by the characteristics of the dialyzer capillary. Theoretically, PSP is subject to significant convective elimination in continuous hemodialysis and the use of hybrid technologies that utilize devices with adsorptive membrane characteristics [46,47]. Its removal occurs primarily during hemofiltration and continuous hemodiafiltration treatments [48].

The clearance capacity of β2-microglobulin (B2M), a middle-molecule uremic toxin, characterizes the type of dialyzer, from low-flux (with clearance of less than 10 mL/min) to so-called super-flux dialyzers (with clearance greater than 70 mL/min). The importance of B2M for the analysis of PSP levels lies in the proximity of their low molecular weights (has 11.8 kDa for B2M, and 13 kDa for PSP, approximately) [49]. Thus, PSP levels decreased using membranes with B2M clearance of ≥50 mL/min, while levels increased using membranes with clearance of <30 mL/min [49]. Furthermore, PSP’s removal using high-flux membranes indicates that its protein binding is negligible [49].

However, blood levels may increase during this extracorporeal therapy. This biomarker can be measured before this procedure to mitigate the effect of HD on its removal [29]. Therefore, some authors argue that the reliability of using PSP in AKI patients with KDIGO 3 requiring renal replacement therapy (RRT) is reduced [15,33].

### 2.11. Prognosis—Survival and Mortality

The use of PSP as a prognostic biomarker in SA-AKI has been proposed. Promising results were demonstrated by Lee et al., who found better performance of PSP compared with APACHE II and SOFA scores as an independent risk factor for death within 28 days, specifically in this group of patients (HR: 3.437; *p* = 0.03) [29]. In the same study, PSP was also found to be superior to CRP and PCT (with AuROC values of 0.765, 0.477, and 0.608, respectively) as a predictor of mortality in patients with SA-AKI.

Hwang and colleagues demonstrated discrepant findings [50], with AuROC values for PSP, APACHE II, and SOFA of 0.636, 0.663, and 0.731, respectively; however, presepsin was considered an independent risk factor for 28-day mortality in the SA-AKI subgroup (HR: 6.868; *p* = 0.005). No superiority of PCT over PSP was described by Ali and colleagues [28], with AUCs of 0.932 and 0.891, respectively (*p* = 0.465), in relation to 28-day mortality.

Although a study revealed that the mortality of patients who recovered from SA-AKI was comparable to that of patients who did not recover [51], biomarkers are a tool of great prognostic value. Han and colleagues’ study cited an optimal cutoff value of greater than 693 pg/mL in the prediction of death, but with a log-rank test with a *p*-value of 0.144 [32].

## 3. Methodology

### 3.1. Study Type

A scoping review was carried out per the PRISMA-ScR (Preferred Reporting Items for Systematic Reviews and Meta-Analysis–Extension for Scoping Reviews) guidelines. This study was protocolled and registered on the Open Science Framework under DOI https://doi.org/10.17605/OSF.IO/Z8U25 and internet archive link https://archive.org/details/osf-registrations-z8u25-v1 accessed on 9 september 2025.

### 3.2. Search Strategy

A search was performed in the PubMed/Medline, Web of Science, Embase, and Scopus databases, using the combination of controlled descriptors (MeSH/DeCS) and keywords: (“presepsin” OR “sCD14-ST” OR “solube CD 14 subtype”) AND (“acute kidney injury” OR “AKI” OR “renal injury”) AND (“sepsis” OR “septic shock”) AND (“sepsis associated acute kidney injury” OR “SA-AKI”), from 2010 to 2025.

### 3.3. Eligibility

#### 3.3.1. Inclusion Criteria

Primary studies were accepted for inclusion in this review, including observational studies, clinical trials, cohort studies, and diagnostic accuracy studies; studies covering adult populations over 18 years of age; and studies in which the full text was available.

#### 3.3.2. Exclusion Criteria

Experimental studies with animals or in vitro experimental models; narrative reviews (used only for bibliographic screening); and articles in which it was not possible to access the full text were excluded from this review.

### 3.4. Selection of Studies

**Figure 1:**
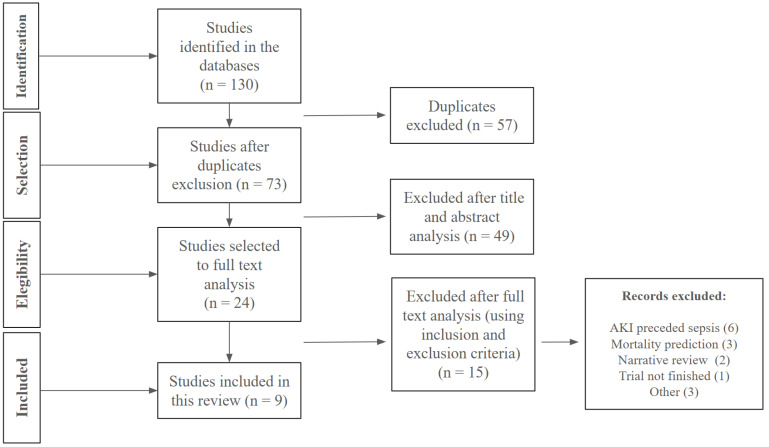
Description of the selection of studies included in this review. AKI: acute kidney injury.

After consulting the databases, the files were forwarded to the selection application RAYYAN^®^ (AI-Powered Systematic Review Management Platform–Qatar Foundation), Cambridge, Massachusetts, USA. This platform organized all articles and eliminated duplicates. Following this step, two reviewers independently selected articles based on title and abstract. In case the reviewers disagreed, a third reader would decide whether to include the article. After completing this selection process, the articles’ full versions were read to review and confirm which of them would be approved for data extraction and analysis. This step is described in Figure 1.

### 3.5. Data Extraction and Analysis

The following data were selected for extraction: authors’ names, year, and country; study design; population; outcomes assessed (AUC, sensitivity, specificity, and mortality); and reported PSP cutoff blood levels. A descriptive analysis was performed, with the narrative synthesis and data presentation shown in Table 1.

### 3.6. Ethical Considerations

This scoping review was conducted using secondary literature; there was no direct involvement of human subjects or primary data collection. Therefore, it was exempt from submission to the Research Ethics Committee, as per CNS Resolution 510/2016 [60]. However, out of respect for ethical principles and academic integrity, the sources were fully cited.

### 3.7. Selected Studies

The following number of studies were selected after searching the databases: PubMed/MEDLINE: 12; Web of Science: 35; Embase: 58; and Scopus: 25. The information is described in Table 2.

## 4. Results

Nine references were selected for analysis [30,52,53,54,55,56,57,58,59], as described in Figure 1 and Table 1. The articles were published between 2016 and 2025, five of which were conducted in Japan, two in South Korea, one in Indonesia, and one in Thailand. Most publications (seven articles) were conducted prospectively, with populations ranging from 56 to 246 patients. The mortality rates in the groups ranged from 14 to 64.29%.

Statistical analysis revealed AUC values ranging from 0.595 to 0.84, with sensitivity values ranging from 0.58 to 0.95 and specificity values ranging from 0.52 to 0.0817. The ideal cutoff values ranged from 572 to 1373 pg/mL. The results are described in Table 1.

## 5. Discussion

PSP has become a promising biomarker in the diagnosis of sepsis because it reflects the activation of monocytes and macrophages in response to bacterial infections, representing the cellular immune response [61]. Impaired renal function is expected to lead to pathophysiological changes in its elimination, limiting its use as a biomarker in pathological conditions that determine renal dysfunction. PSP levels have long been correlated with renal function, but their reliability as a biomarker in clinical conditions that alter it, such as sepsis, has been questioned. PSP elimination by the kidneys means that its levels in non-septic kidney injury models are influenced by impaired kidney function, so its rise cannot be solely relied upon to differentiate sepsis from idiopathic or multifactorial severe kidney damage in these patients. Thus, levels will likely be higher simply due to reduced renal excretion, regardless of an underlying infection. Likewise, levels will increase in non-septic inflammation [16] or in chronic kidney disease patients, in which PSP cutoff levels require careful consideration [15]. In a study conducted over a decade ago by Nakamura et al., PSP proved to be a reliable biomarker in early-stage AKI, but not in advanced stages, where there was no statistically significant difference between the sepsis and non-sepsis groups (*p* = 0.3) [16].

Furthermore, in individuals whose AKI progresses to the end stage, requiring hemodialysis, an extracorporeal form of RRT also affects plasma PSP levels. PSP levels may increase due to the activation of neutrophils and monocytes and subsequent release by the latter [52]. PSP levels are increased in hemodialysis patients without any evidence of sepsis compared with healthy individuals [62]. Conversely, PSP can be removed using different modalities of renal replacement therapy, as its molecule is subject to significant convective elimination [47], reducing its plasma levels [54,62]. Thus, patients with AKI, especially those on HD, have different confounding factors for measuring plasma PSP levels. The innate and adaptive immune responses, fundamental in the defense against pathogens, stimulate organ damage via imbalances in coagulation and cytokine cascades, endothelial dysfunction, and proinflammatory mechanisms [63]. Life-threatening organ dysfunction is involved in sepsis [64], where renal impairment is common via different injury mechanisms [23]. In this context, the coexistence of sepsis and AKI has prompted the recommendation of a specific nomenclature that would consensually define the latter [23]. The concept of SA-AKI, representing the coexistence of AKI and sepsis, emerges in a scenario where efforts are being made to prioritize this complication of septic syndrome, a leading event in the clinical deterioration of critically ill patients. Despite notable advances in prognosis, there has been little progress in therapeutic proposals in recent decades [65]. This is perhaps due to the oversimplification of a heterogeneous condition, caused both by the direct effect of infection and the host’s response to its presence [23], stemming from rigid clinical criteria, such as the KDIGO [66]. Therefore, clinical trials are awaited to indicate potential treatment strategies, which depend on understanding the intricate pathophysiology of SA-AKI [24]. Phenotyping SA-AKI subtypes would be an alternative in the search for targeted therapies, better suited to each etiology [65].

PSP is beneficial in patients with SA-AKI because it is produced in response to bacterial infections [35], with possible superiority in diagnostic accuracy, being considered a promising prognostic biomarker in SA-AKI by Lee et al. [29]. However, different cutoff points for the diagnosis of sepsis are suggested, depending on the estimated GFR and AKI severity, which could be detrimental to the diagnosis.

In the context of SA-AKI, PSP has a critical advantage over PCT due to its specificity to infection, usefulness in patients with recurrent sepsis, where it would prevent the discontinuation of antibiotic therapy [36], and moderate diagnostic value in discriminating AKI [32], being considered a satisfactory biomarker in this scenario.

However, its use in critically ill patients is controversial. In addition to renal dysfunction, which is prevalent in that population and a confounding factor in PSP blood levels, the concomitant use of medications can, theoretically, interfere with PSP kinetics. For example, corticosteroids appear to be harmless to PSP at low doses [67], but reduce CD14 expression in monocytes [68], and propofol, an intravenous anesthetic widely used in critically ill patients, reduces CD14 expression in the blood [69], potentially leading to decreased expression of inflammatory biomarkers [70]. The influence of these and other medications on PSP remains unknown, despite the clear relevance of the topic.

Recommendations for the use of PSP as a diagnostic adjunct in sepsis remain limited, with no indication for routine use yet defined. The Surviving Sepsis Campaign (SSC), in its most recent version, recommends only procalcitonin as a biomarker, solely as an aid in the decision to discontinue antibiotic therapy [71]. However, PSP has no diagnostic function in this global guideline, being recommended only as a supplement to clinical observation, as described in the 2020 Japanese Clinical Guidelines for the Management of Sepsis and Septic Shock [14].

This review revealed that presepsin had good diagnostic or prognostic accuracy in Takahashi and collaborators’ study (with an AUC of 0.84) [52], moderate in five studies (with AUC values of 0.793 [56]; 0.77 [53]; 0.73 [54]; 0.71 [58]; and 0.70 [55], respectively), and low in two publications (with AUC values of 0.69 [57] and 0.595 [30], respectively). Only one of the studies did not provide this information [59].

The selected studies revealed some nuances regarding the use of PSP in SA-AKI. Serum creatinine levels, when assessed in the first two days of ICU admission, fail to predict mortality at 28, 60, 90, or 180 days (*p* = 0.3, *p* = 0.37, *p* = 0.44, and *p* = 0.4, respectively) [53]. Takahashi and colleagues indicate that blood levels of this biomarker can increase during hemodialysis due to monocyte activation, leading to the release of PSS by these elements [52]. Kim and colleagues [55] claimed that the ideal PSP cutoff value was > 572 pg/mL to predict the diagnosis of SA-AKI (with a sensitivity of 77% and a specificity of 81.7%). Regarding prognostic accuracy, the superiority of the values identified on the first day (with AUC, sensitivity, and specificity values of 0.69, 82%, and 52%, respectively) may suggest the early use of PSP to rule out progression to SA-AKI [57].

When considering the results obtained in the studies included in this review, we observed that PSP was considered a good predictor of SA-AKI and initiation of HD when measured on the second day of ICU admission [54], revealing the importance of sequential measurement and evaluation of its dynamic behavior. A presepsin level cutoff point of ≥ 795 pg/mL would be associated with progression to SA-AKI [59]. Favorable results were also found by Kim and colleagues [56], with an AUC of 0.793 (95% CI: 0.729–0.848; *p* < 0.001) for the prediction of SA-AKI. When used in individuals over 75 years of age, PSP has shown greater predictive value for progression to SA-AKI than in patients under this age, especially in samples collected on the second day of hospitalization (AUC: 0.73 and *p* = 0.024; AUC: 0.69 and *p* = 0.058, respectively) [58].

The evaluation of the studies revealed many inconsistencies due to heterogeneity in values defined as borderline, usually calculated via ROC analysis to maximize the biomarker’s diagnostic performance, or obtained using the Youden index. No studies have defined the existence or specific values for viral, bacterial, fungal, and parasitic etiologies. Moreover, there is no agreement on borderline PSP blood values for the diagnosis or prognosis of septic syndrome or SA-AKI, noting that diagnostic accuracy varies with these values assigned in each study.

The variation in the definition of the control group may also be a methodological weakness, given that the group is sometimes composed of healthy individuals or those with non-infectious SIRS [72]. However, this dissimilarity between values and lack of standardization may reveal the clinical heterogeneity of the groups analyzed.

Another possible source of confusion is the type of sample submitted for analysis, whether whole blood or plasma, considering that no study has correlated the values obtained from both specimens. The methodology widely used in the studies analyzed (CLEIA) allows the use of whole blood, plasma, or thawed plasma [43]. Although some subgroup analyses suggest better specificity results with the use of whole blood [72], there is no reported correlation (linear or otherwise) between the values obtained with the different methods.

## 6. Future Prospects

Future research should address unfinished business via a better understanding of the pathophysiology of SA-AKI and the predictive mechanism of presepsin in relation to the severity of this condition. Research becomes particularly challenging in emergency and intensive care settings, where organ dysfunction, use of sedative and vasopressor medications, mechanical ventilation, and shock are combined.

A personalized approach to septic syndrome, encompassing metabolomic and transcriptomic analyses, as well as phenotyping and definition of SA-AKI subgroups, suggesting a customized therapeutic approach for each individual, could be part of the unique treatment provided by precision medicine. Such genomic tools would facilitate the use of multi-biomarker panels, a promising model for diagnostic anticipation and therapeutic accuracy [73].

## 7. Conclusions

This review suggests that PSP has proven to be a biomarker with good diagnostic and prognostic accuracy in AKI, although the data were gathered from observations of small patient groups. The scarcity of data from randomized clinical trials, systematic reviews, and meta-analyses indicates that this topic requires further attention and improvement, particularly for providing more reliable threshold values for sepsis diagnosis and prognosis.

## Figures and Tables

**Table 1:** Characteristics of selected studies.

Number	Authors	Year	Country	Type	Population	Outcomes				
						Sensibility	Specificity	AUC	Mortality	Cutoff (pg/mL)
1	Takahashi, G., Shibata, S., Fukui, Y., Okamura, Y. & Inoue, Y. [52]	2016	Japan	Retrospective	91	0.69	0.79	0.84	-	891
2	Shimoyama, Y., Umegaki, O., Kadono, N. & Minami, T. [53]	2021	Japan	Prospective	83	0.82	0.77	0.77	-	1373
3	Shimoyama, Y., Umegaki, O., Kadono, N. & Minami, T. [54]	2021	Japan	Prospective	83	0.82	0.59	0.73	31.3%	708
4	Kim, S.Y., Hong, D.Y., Kim, J.W., Park, S.O., Lee, K.R. & Baek, K.J. [55]	2022	Korea	Retrospective	151	0.66	0.691	0.700	12.6%	572
5	Kim, S.Y., Hong, D.Y., Lee, K.R., Paik, J.H. & Jung, H.M. [56]	2022	Korea	Prospective	193	0.77	0.817	0.793	14%	572
6	Shimoyama, Y., Umegaki, O., Kadono, N. & Minami, T. [57]	2022	Japan	Prospective	59	0.82	0.52	0.69	42%	708
7	Shimoyama, Y., Kadono, N. & Umegaki, O. [58]	2024	Japan	Prospective	83	0.95	0.53	0.71	31.3%	627
8	Isaranuwatchai, S., Sophonphan, J., Voharnsuchon, P., Thong-on, K. & Sri-on, J. [59]	2024	Thailand	Prospective	246	-	-	-	24%	795
9	Puspitasari, S., Semedi, B.P., Rehatta, N.M., Maulydia, M. & Purnomo, W. [30]	2025	Indonésia	Prospective	56	0.581	0.625	0.595	64.29%	-

**Table 2:** Database search results.

Database	PubMed	Web of Science	Embase	Scopus
Number of references	12	35	58	25

## Data Availability

No new data were created or analyzed in this study. Data sharing is not applicable to this article.
